# Ion Release and Apatite Formation of Resin Based Pit and Fissure Sealants Containing 45S5 Bioactive Glass

**DOI:** 10.3390/polym16131855

**Published:** 2024-06-28

**Authors:** Ji-Won Choi, A Ruem Han, Song-Yi Yang

**Affiliations:** 1 Department of Dental Hygiene, Kyungdong University, Wonju-si 26495, Republic of Korea; 2 Department and Research Institute of Dental Biomaterials and Bioengineering, Yonsei University College of Dentistry, Seoul 03722, Republic of Korea; 3 Department of Dental Hygiene, Konyang University, Daejeon 35365, Republic of Korea

**Keywords:** pit and fissure sealant, 45S5 bioactive glass, apatite formation, ion release, pH variation

## Abstract

The purpose of this study was to evaluate a resin based pit and fissure sealant containing 45S5 bioactive glass (BAG) by examining its ion release, pH variation, and apatite-forming properties. To prepare the experimental materials, 45S5 BAG, used as a filler, was incorporated into the light curable resin matrix at concentrations of 0 (control), 12.5, 37.5, and 50.0 wt.%. Ion release, pH variation, and apatite formation (Raman spectrometer and scanning electron microscopy-energy-dispersive X-ray spectrometry measurements) were performed. While no ions were released from the control group, the experimental groups containing 45S5 BAG showed an increased release of Ca and P ions with increasing amounts of 45S5 BAG (*p* < 0.05). The pH of the experimental group remained high and was significantly different from the control group (*p* < 0.05). Unlike the control group, it was confirmed that the apatite peak was formed in the 50.0 wt.% BAG group for 90 days, and the apatite layer consisting of Ca and P was deposited on the surface. Thus, a resin based pit and fissure sealant containing 45S5 BAG is a promising material for preventing secondary caries by releasing ions and forming apatite.

## 1. Introduction

Dental caries is the most common oral disease and an important public health issue with negative outcomes [1]. Untreated dental caries, especially during childhood, can seriously harm a person’s overall health and quality of life [2,3]. It can also cause disruptions in eating patterns, hospitalization, and slowed growth [4,5]. According to a study, 80–90% and 44% of all carious lesions in permanent and primary teeth, respectively, are caused by pit and fissure caries [6], which is because the occlusal surfaces are morphologically irregular and complex [7].

Pit and fissure sealants are frequently used to prevent pit and fissure caries and to arrest the incipient caries on the occlusal surfaces by forming a physical barrier [8,9]. Previous research has shown that the effectiveness of pit and fissure sealants in preventing or arresting caries [10,11]. Pit and fissure sealants are designed to fill the pits and fissures on tooth surfaces; however, if not applied thoroughly to reach the bottom, this can result in the formation of spaces underneath the polymerized sealant [12]. Owing to microleakages or partial detachments from the tooth, secondary or recurrent caries can often occur between the enamel surface and the pit and fissure sealant [13]. 

To overcome these limitations, pit and fissure sealants with bioactive components have been developed [13,14,15,16,17,18]. However, many studies have focused only on their anti-bacterial or anti-fouling effects to inhibit dental caries [14,15,16,17]. Other research has concentrated on the potential of pit and fissure sealants with bioactive components to release hydroxide ions to neutralize acids [13,18]. However, since the primary role of pit and fissure sealants is to form a physical barrier, the ability of the material to remineralize the space between the structure of the tooth and the material for prevention by forming apatite over the long term is also important for the treatment outcome [19]. 

Hench et al. first synthesized 45S5 bioactive glass (BAG) in 1971, and it has been used in clinical settings since 1985 [20]. The outstanding mechanical and biological qualities of 45S5, which is composed of silica, sodium, calcium, and phosphorus oxides, have led to the widespread usage of bone substitutes in bioengineering [21]. Furthermore, the potential of 45S5 BAG as a desensitizer has been reported owing to its ability to precipitate apatite into dentinal tubules [22]. Similarly, orthodontic adhesive and tooth whitening products that contain 45S5 BAG can be successfully buffered in a low pH environment [23,24]. According to a study in which 45S5 BAG was applied as a pit and fissure sealant in a cariogenic environment, the surface roughness of the enamel surface was not adversely affected by an increase in Vickers hardness [25]. Therefore, 45S5 BAG has the potential to promote mineralization when applied to deep pits and fissures.

To our knowledge, there has been no detailed study on the long term surface and cross sectional analyses of the apatite forming properties of resin based pit and fissure sealants with 45S5 BAG. Accordingly, there is a need for an analysis that quantitatively and qualitatively demonstrates that apatite is formed by 45S5 BAG when 45S5 BAG is present in the resin based pit and fissure sealant.

Thus, the purpose of this study was to evaluate a resin based pit and fissure sealant containing 45S5 BAG by examining its ion release, pH variation, and apatite formation properties. The null hypothesis of this study was that the ion release, pH variation, and the apatite forming ability of the pit and fissure sealants containing 45S5 BAG would not differ significantly from those of the pit and fissure sealants without the 45S5 BAG filler.

## 2. Materials and Methods

### 2.1. Synthesis of 45S5 BAG Powder

To obtain a composition similar to that of 45S5 BAG with weight percentages (wt.%) of 45.0 SiO_2_, 24.5 CaO, 24.5 Na_2_O, 6.0 P_2_O_5_, powders of SiO_2_ (Junsei Chemical Co., Tokyo, Japan), Na_2_CO_3_ (Duksan Reagents, Gyeonggi-do, Republic of Korea), CaCO_3_ (Samchun Pure Chemicals Co., Gyeonggi-do, Republic of Korea), and P_2_O_5_ (Sigma-Aldrich, St. Louis, MO, USA) were measured. The combined powders were melted at 1400 °C for 4 h in a Pt crucible and subsequently quenched into a graphite plate mold at 23 ± 2 °C. Using a mortar and pestle, 45S5 BAG produced from the melt was crushed and filtered through a 500-mesh sieve. The morphology and chemical composition of the particles coated with Pt were observed using scanning electron microscopy and energy-dispersive X-ray spectrometry (SEM-EDS; JEOL-7610F-plus, JEOL Ltd., Peabody, MA, USA) at a magnification of 900×.

### 2.2. Preparation of Resin Matrix

On the basis of the information regarding the composition of commercially available pit and fissure sealants, a resin matrix was created using a mixture of 49.5 wt.% bisphenol A glycerolate dimethacrylate (Sigma-Aldrich, St. Louis, MO, USA) and 49.5 wt.% triethylene glycol dimethacrylate (Sigma-Aldrich, St. Louis, MO, USA) in a 1:1 mass ratio. Subsequently, 0.3 wt.% camphorquinone (Sigma-Aldrich, St. Louis, MO, USA) and 0.6 wt.% 2-(dimethylamino)ethyl methacrylate (Sigma-Aldrich, St. Louis, MO, USA) were added as a photoinitiator and an accelerator, respectively. In different proportions, 45S5 BAG and 180 ± 30 nm of silanized glass particles were incorporated to the resin matrix (Table 1).

### 2.3. Ion Release and pH Variation

To fabricate the specimens for the analysis of ion release and pH variation, the unpolymerized experimental material was placed into a mold with a diameter of 10 mm and a height of 1 mm. Each specimen was then uniformly cured using a light emitting diode curing unit (Elipar S10; 3M ESPE Co., Seefeld, Germany) at a light intensity of 1200 mW/cm^2^ for 20 s on each side. After separating the polymerized specimen from the mold, the specimen was stored in distilled water (DW; JW Pharmaceutical, Chungcheongnam-do, Republic of Korea) according to the extraction ratio (3 cm^2^/mL) described in ISO 10993-12 (2021) [26]. Each specimen was fully submerged in an individual conical tube filled with the DW, ensuring that all surfaces were exposed to the solution. The immersion solution was replaced weekly. The release of Ca and P ions from the DW-immersed specimens was measured at 1, 7, 14, 30, 60, and 90 days using inductively coupled plasma optical emission spectrometry (ICP-OES, Optima 8300; PerkinElmer, Waltham, MA, USA). The solutions used to test the ion release were also used to test the pH variation. The pH variation in the DW-immersed specimens was measured at 1, 7, 14, 30, 60, and 90 days using a calibrated pH meter (Orion 4 Star; Thermo Fisher Scientific Inc., Waltham, MA, USA) with buffer solutions of pH 4.01, 7.0, and 10.01. All ion releases and pH measurements were repeated six times, and the mean and standard deviation were determined.

### 2.4. Apatite Forming Properties

Six disk shaped specimens (10.0 × 1.0 mm) were prepared for each group as previously described in ‘Ion release and pH variation’. To simulate oral conditions, artificial saliva containing 0.4411 g CaCl_2_·2H_2_O (Sigma-Aldrich, St. Louis, MO, USA), 0.245 g KH_2_PO_4_ (Sigma-Aldrich, St. Louis, MO, USA), and 800 mL of DW was used as an immersion solution. In addition, 0.5 M KOH (Duksan Reagents, Gyeonggi-do, Republic of Korea) was used to adjust the pH of the artificial saliva to 7.0 [27]. All the specimens were immersed in the artificial saliva according to the extraction ratio (3 cm^2^/mL) described in ISO 10993-12 (2021) [26] and stored at 37 ± 1 °C for 14, 30, 60, and 90 days. Each specimen was fully submerged in an individual conical tube filled with artificial saliva, ensuring that all surfaces were exposed to the solution. The artificial saliva was refreshed weekly. The precipitates present on the surface of the specimens were carefully scraped, after which Raman spectrometry was performed. Raman spectroscopy was used to confirm the properties of the molecules formed on the specimens under 50× using a Raman spectrometer (LabRam Aramis, Horriba Jobin Yvon, France), having an opening of 50 µm, laser wavelength of 532 nm, and Raman shift ranging from 200 to 1400 cm^−1^ (*n* = 4) [28]. It was equipped with an ND:Yag laser (λ = 532 nm, a laser power of 50 mW) as an excitation source.

In addition, to observe the cross section and surface of the specimen, SEM-EDS (Merin, Carl Zeiss, Oberkochen, Germany) was performed under a 500× magnification and a 15 kV accelerating voltage (*n* = 2). The SEM-EDS specimens were sputter coated with Pt before the analysis using an ion sputterer (Leica EM ACE 600; Leica, Wien, Austria).

### 2.5. Statistical Analysis

The one-way analysis of variance method and Tukey’s post hoc test were used to analyze the data on ion release and pH variation using the SPSS 25 (IBM Co., Armonk, NY, USA) program. The level of significance was set at *p* < 0.05.

## 3. Results

### 3.1. Powder Characterization of 45S5 BAG

**Figure 1:**
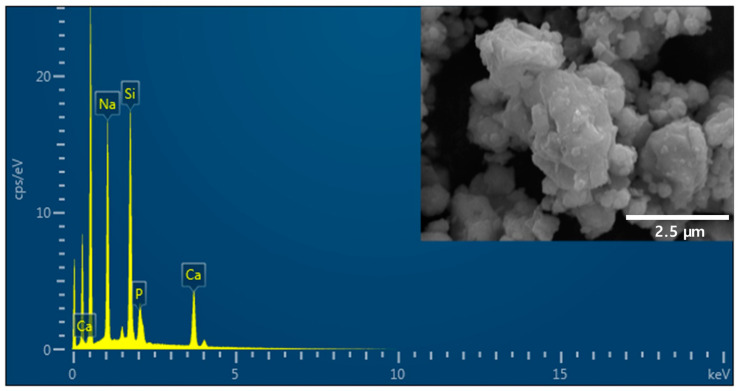
Scanning electron microscopy and energy-dispersive X-ray microscopy (SEM-EDS) images of 45S5 bioactive glass (BAG) powder. The SEM image in the top right shows that the 45S5 BAG powder exhibits agglomerates with irregular and sharp edges.

Figure 1 shows the morphology and chemical composition of 45S5 BAG powder observed at 900× magnification. The 45S5 BAG powder exhibited agglomerates with irregular and sharp edges. In addition, EDS was used to detect the main constituents of Si, Na, Ca, and P. These configurations match the composition of 45S5 BAG.

### 3.2. Ca and P Ion Release

**Figure 2:**
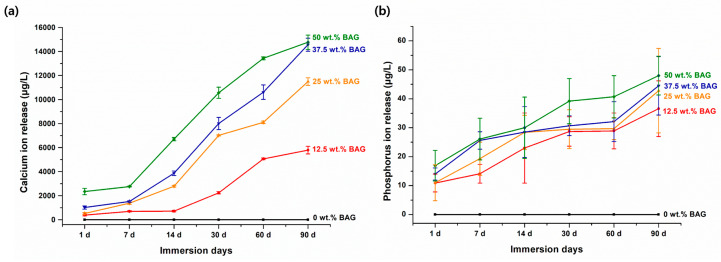
Results of Ca (**a**) and P (**b**) ion release. Each value represents the mean of six repeated measurements, and the error bars show the standard deviation of the mean values. BAG, bioactive glass.

Figure 2 shows the release of Ca and P ions from each specimen. The results indicated an increase in the release of these ions with an increase in the amount of 45S5 BAG. In addition, unlike in the experimental groups, the release of Ca and P ions was not observed in the control group, in which 45S5 BAG was not added. The maximum release of Ca ions occurred from the 50 wt.% BAG group, which was 2.5 times higher than that of the 12.5 wt.% BAG group (*p* < 0.001). In particular, for the 50 wt.% BAG group, we confirmed that the amount of Ca ion release was approximately six times higher on the 90th day of immersion than on the 1st day. The final Ca ion concentrations of the 12.5, 25, 37.5, and 50 wt.% BAG groups were 5790.6 ± 316.6, 11,495.5 ± 313.1, 14,563.7 ± 541.2, and 14,765.3 ± 594.5 μg/L, respectively. Similar to the results of the Ca ion release, the maximum release of P ions was from the 50 wt.% BAG group, which was 1.3 times higher than that of the 12.5 wt.% BAG group (*p* < 0.001). The final P ion concentrations of the 12.5, 25, 37.5, and 50 wt.% BAG groups were 36.6 ± 9.6, 42.8 ± 14.6, 44.5 ± 10.1, and 48.0 ± 6.7 μg/L, respectively. In particular, there were no significant differences in the release of Ca and P ions between the 50 and 37.5 wt.% BAG groups at the final point (*p* > 0.05).

### 3.3. pH Variation

**Figure 3:**
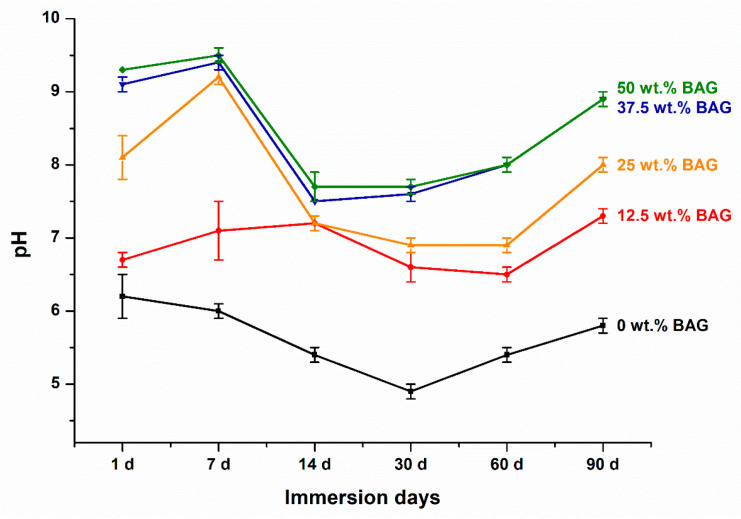
Results of the pH variation. Each value represents the mean of six repeated measurements, and the error bars show the standard deviation of the mean values. BAG, bioactive glass.

Figure 3 shows the pH variation over 90 days for each specimen. At all time points, a higher pH was observed as the amount of 45S5 BAG increased (*p* < 0.001). However, after 90 days of immersion, there was no significant difference in pH between the 37.5 and 50 wt.% BAG groups (*p* > 0.05). The experimental group including 45S5 BAG showed a pH of 6.5 or higher during all periods, whereas the control group without 45S5 BAG showed the lowest value of pH at 4.9 on the 30th day of immersion, subsequently reaching a pH of 5.8 on the 90th day of immersion. However, this was significantly lower than that in the experimental group (*p* < 0.05). The final pH of the 0, 12.5, 25, 37.5, and 50 wt.% BAG groups were 5.8 ± 0.1, 7.3 ± 0.1, 8.0 ± 0.1, 8.9 ± 0.1, and 8.9 ± 0.1, respectively.

### 3.4. Analysis of Apatite Formation with Raman Spectroscopy

**Figure 4:**
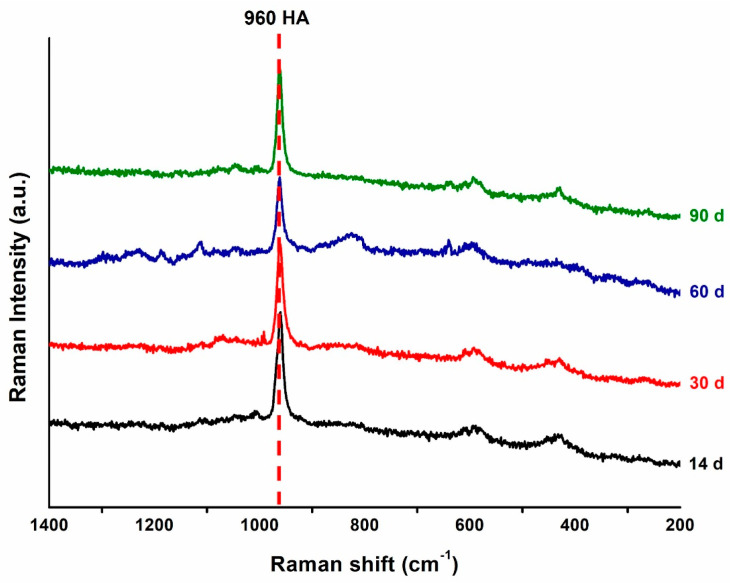
Representative Raman spectra of the precipitates on the 50 wt.% bioactive glass (BAG) specimen surface after 14 (black), 30 (red), 60 (blue), and 90 (green) days of storage in the artificial saliva. HA, hydroxyapatite.

In this study, surface precipitates observed on the specimens were subjected to Raman spectroscopy analysis based on their presence and detectability. The surface precipitates produced after immersing the specimens of the 50 wt.% BAG group for 14, 30, 60, and 90 days in artificial saliva are shown in Figure 4 as representative Raman spectra. The 0 wt.% BAG group immersed in the artificial saliva was excluded from the Raman spectroscopy analysis because no precipitate formed on its surface. In contrast, after immersing 50 wt.% BAG in the artificial saliva for 90 days and analyzing the precipitate at 960 cm^−1^, a peak of hydroxyapatite with similar intensity was confirmed for a long period.

### 3.5. Analysis of Apatite Formation with SEM-EDS

**Figure 5:**
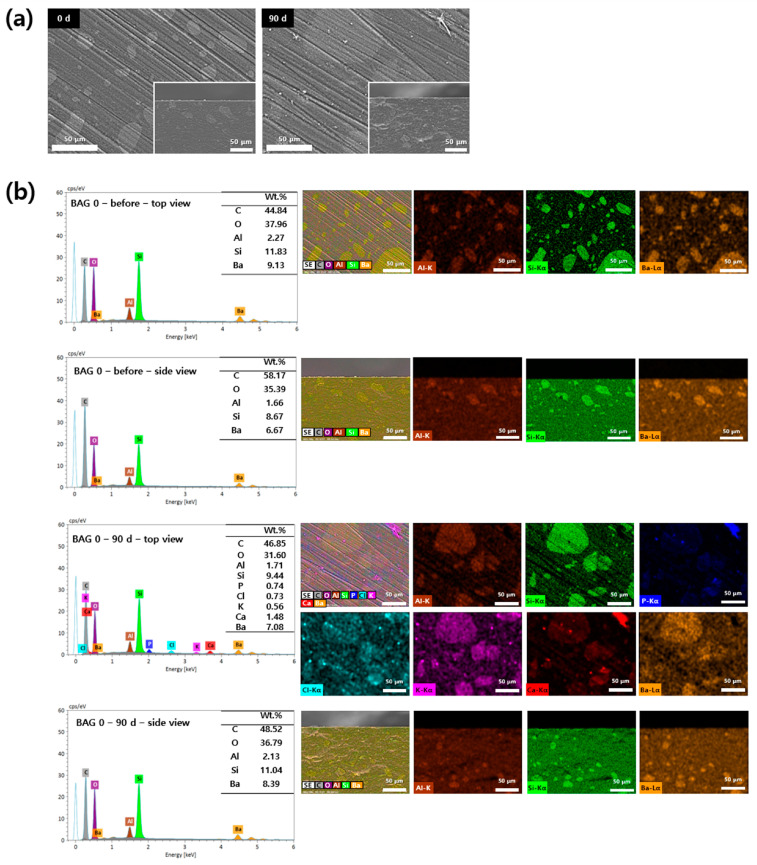
(**a**) Representative scanning electron microscopy (SEM) images of the 0 wt.% bioactive glass (BAG) immersed in the artificial saliva for 0 (left) and 90 (right) days at magnifications of 500×. The main images show the top view of the specimen surfaces, and the lower-right smaller images show the side view. (**b**) Representative SEM-energy-dispersive X-ray spectrometry (SEM-EDS) images of the top and side views of the surfaces of 0 wt.% BAG immersed in the artificial saliva for 0 and 90 days at magnifications of 500×. EDS results indicate Al as brown, Si as green, P as blue, Cl as sky blue, K as purple, Ca as red, and Ba as orange.

**Figure 6:**
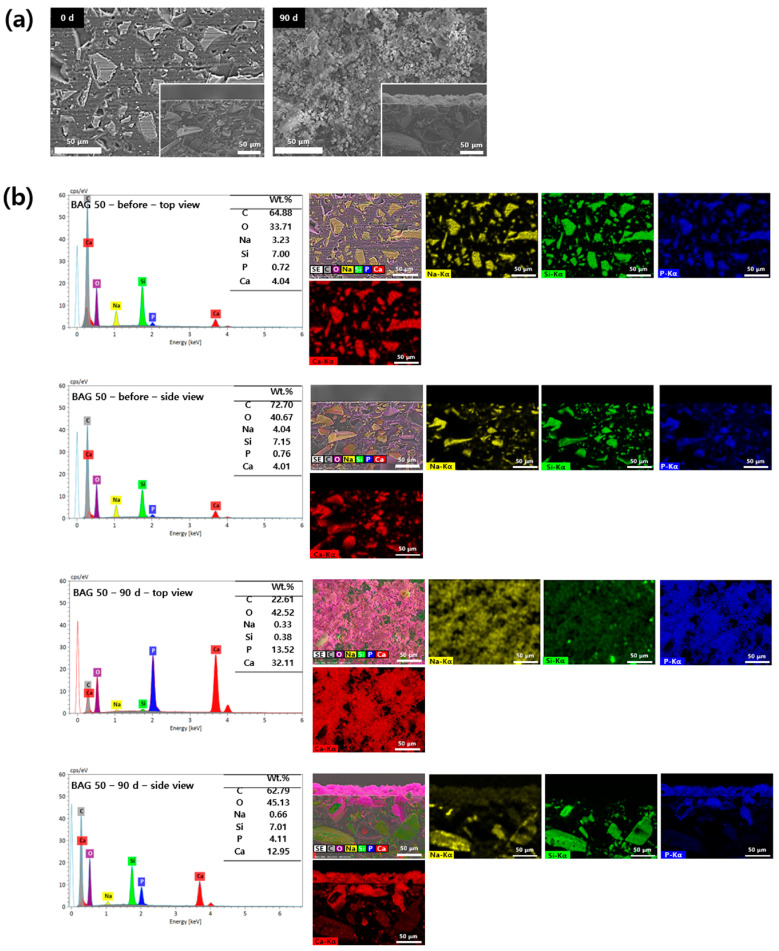
(**a**) Representative scanning electron microscopy (SEM) images of the 50 wt.% bioactive glass (BAG) immersed in the artificial saliva for 0 (left) and 90 (right) days at magnifications of 500×. The main images show the top view of the specimen surfaces, and the lower-right smaller images show the side view. (**b**) Representative SEM-energy-dispersive X-ray spectrometry (SEM-EDS) images of the top and side views of the surfaces of 50 wt.% BAG immersed in the artificial saliva for 0 and 90 days at magnifications of 500×. EDS results indicate Na as yellow, Si as green, P as blue, and Ca as red.

Surface precipitates observed on the specimens were subjected to SEM-EDS analysis based on their presence and detectability. The 0 and 50 wt.% BAG specimens are represented by SEM-EDS images in Figure 5 and Figure 6. In the surface image of the 0 wt.% BAG specimen, the polished aspect was still observed even after being submerged for 90 days in the artificial saliva, and we confirmed the absence of deposits on the surface in the cross sectional image (Figure 5a). In contrast, in the surface image of the 50 wt.% BAG specimen, no polished surface was observed after immersion in the artificial saliva for 90 days, and the accumulation of sediment on the surface was confirmed in the cross sectional image (Figure 6a). Through SEM-EDS analysis of the 0 wt.% BAG specimen submerged for 90 days in the artificial saliva, we observed the presence of Al, Si, and Ba as components of the filler (Figure 5b). In contrast, for the 50 wt.% BAG group Ca, P, and Na were found in addition to Al, Si, and Ba (Figure 6b). In particular, on top of the 50 wt.% BAG specimen, as seen in the image of the cross section, was a sediment layer that contained Ca and P.

## 4. Discussion

In the field of bone grafts and tissue scaffolds, 45S5 BAG has been extensively studied because of its ability to promote osteoinduction, osteoconduction, osseointegration, and angiogenesis [29,30,31]. In addition, 45S5 BAG has also been used as a component of composite resins, adhesive cements, and temporary filling materials owing to its desirable property of inducing remineralization [16,32,33]. Although the bioactivity and biocompatibility effects of 45S5 BAG on dental materials are well known, there are only a few comprehensive studies on long term self-sealing restorative materials to minimize or completely seal any microleakage between the cavity walls and filling materials. Therefore, in this study, ion release, pH variation, and apatite forming characteristics were evaluated by developing a pit and fissure sealant comprising 45S5 BAG, which had a self-marginal sealing potential by forming hydroxyapatite.

First, 45S5 BAG was synthesized, and we confirmed through SEM-EDS that this was properly performed. Specifically, an agglomerated appearance of 45S5 BAG with irregular and sharp edges was observed using SEM. In addition, the main components of 45S5 BAG—Si, Na, Ca, and P—were detected using EDS. In terms of structure and composition, the prepared 45S5 BAG was similar to 45S5 Bioglass, which is widely available [34].

As the concentration of 45S5 BAG filler was increased, there was a corresponding enhancement in the release of calcium and phosphorus ions, while the pH levels remained elevated. Furthermore, distinct from the control group that contained no 45S5 BAG filler, the group with 50.0 wt.% BAG exhibited a prominent hydroxyapatite formation peak within 90 days. This was evidenced by the precipitation of hydroxyapatite layers, composed of calcium and phosphorus, on both the surface and cross sectional images. Consequently, the null hypothesis-that the ion release, pH variation, and apatite forming ability of the pit and fissure sealants containing 45S5 BAG would not significantly differ from those without 45S5 BAG filler-was rejected.

The ion release studies revealed that the experimental groups’ released Ca and P ion concentrations were significantly higher than those of the control group. The amount of Ca ions released was approximately six times higher on day 90 of immersion than on day 1 in the 50 wt.% BAG group. In addition, the 50 wt.% BAG group had the highest release of P ion, which was 1.3 times higher than that in the 12.5 wt.% BAG group. These findings may be related to the detection of Ca and P ions when the components of 45S5 BAG powder were verified using EDS. In other words, more Ca and P ions can be released if the pit and fissure sealants contain higher amounts of 45S5 BAG. We used DW as the leaching solution to evaluate the material’s characteristics purely, without the presence of various components such as calcium chloride dihydrate, potassium dihydrogen phosphate, and potassium hydroxide found in artificial saliva [27]. Therefore, the use of DW implies that the release of Ca and P ions is attributed solely to the intrinsic properties of the material, without interactions with the various components present in artificial saliva.

During all periods, a higher pH was maintained as the 45S5 BAG concentration increased. In contrast to the control group, which did not contain 45S5 BAG, the experimental groups consistently displayed a pH of 6.5 or greater. According to a previous study, 45S5 BAG showed a series of reactions that included the release of soluble alkali ions, thereby increasing the pH level [35]. In addition, this also demonstrates the acid neutralizing effect of 45S5 BAG [24]. Our results showed that the pit and fissure sealant containing 45S5 BAG increased the pH in proportion to the amount of 45S5 BAG added. A previous study showed that a pH higher than 5.5 can prevent tooth mineral damage and neutralize local biofilm acids [36]. On this basis, our findings suggest that the long term inhibition of biofilm formation can prevent caries while minimizing the mineral loss of enamel. Excluding the control group, the pH results exhibit an initial upward trend for 7 days, followed by a downward trend until day 14. However, despite this, it indicates that by the final time point at 90 days, it returns to an upward trend, signifying the ability to maintain an alkaline environment. Such pH fluctuation patterns are similar to the findings in a previous study. In prior research, resin monomers based on BisGMA/TEGDMA components showed a decreasing trend within the first 7 days followed by an increasing trend up to day 42. However, it is worth noting that while the previous study used 0.9% saline as the leaching solution, our study utilized DW, potentially leading to differences in the leaching kinetics from resin monomers [37]. To observe the pH variation in the leaching solution of the materials, we used DW. As previously mentioned, artificial saliva contains compounds like calcium chloride dihydrate and potassium hydroxide, which could potentially interact with the ions released from the material and affect the pH readings, leading to inaccurate results. Therefore, it might be difficult to determine whether the obtained results are due to the artificial saliva or the intrinsic properties of the material. Consequently, based on the results mentioned above, even after immersing each specimen in DW for 90 days, the alkaline properties of the material remain evident, with a pH of above 7.

For the 50 wt.% BAG group specimens, the Raman analysis was performed to confirm differences in the physicochemical properties of the precipitate layer on the surface. We confirmed that hydroxyapatite was precipitated on the surface of the 50 wt.% BAG specimen over a long period while being immersed in the artificial saliva, which support the SEM-EDS results confirmed on the surface and cross section images of the specimen. The primary mineral in enamel is hydroxyapatite, which is also the most crucial element in remineralization because of its chemical similarity to human tissue [38]. Therefore, pit and fissure sealant containing 50 wt.% BAG can form hydroxyapatite over a long time, which can positively affect the remineralization of enamel. In multiple studies, the formation of hydroxyapatite has been assessed by focusing on the 960 cm^−1^ peak [39,40]. Therefore, in this study, we also emphasized the analysis of this peak. We were able to confirm the presence of a weak peak commonly observed in all periods at peaks 433 and 579 cm^−1^ in our study results. This peak is associated with phosphate ions and is consistent with the findings of previous research [41,42]. 

In this study, SEM-EDS was performed to analyze the degree of precipitate formation and composition on the surface and cross section of the specimen. Consequently, the specimens of the 50 wt.% BAG group had precipitates on the surface and in the cross-section when compared to those from the control group. More specifically, after immersing in artificial saliva for 90 days, SEM-EDS analysis of the cross section revealed the presence of red precipitates indicative of calcium and blue precipitates indicative of phosphorus on the specimen surface. This observation was consistent even from the top view of the specimen, indicating that the surface was covered with deposits of calcium and phosphorus compounds. A comparison with the SEM-EDS pattern of the 0 day (before) specimen, which was not immersed in artificial saliva, highlights a significant quantitative difference in the accumulated deposits. This indicated that the precipitates composed of hydroxyapatite produced by 45S5 BAG accumulated at the interface over time, possibly even filling the microleakage [43]. Thus, aggregated hydroxyapatite can prevent secondary caries from developing. 

The ability of the resin based pit and fissure sealant containing 45S5 BAG to generate hydroxyapatite, as evidenced by SEM-EDS and Raman spectroscopy, demonstrates its potential to self-seal in the presence of microleakage between the tooth structure and material. This study provides substantial information suggesting that at least 50 wt.% of 45S5 BAG in the resin matrix is necessary for apatite formation. However, increasing the 45S5 BAG content beyond 50 wt.% without compromising the flowability of the pit and fissure sealant is a critical point for future research [44]. Further studies are needed to explore methods of increasing the BAG content while maintaining or improving the flow properties of the material, possibly by modifying the composition of the resin matrix to support higher filler loading. 

On the other hand, a limitation of this study is that some experiments were conducted under conditions that did not fully simulate the complex oral environment. Therefore, for further clinical use, the properties of the resin based pits and fissure sealants containing 45S5 BAG need to be validated in experimental conditions similar to the oral environment or in vivo tests. Furthermore, further evaluation is required to monitor pH changes over an extended period to determine whether the pH value continues to increase, decrease, or reach a saturation point. Additionally, comprehensive assessment is necessary to understand the long term effects of ion release on the mechanical properties of the material.

## 5. Conclusions

In this study, a resin based pit and fissure sealant containing 45S5 BAG, which can react with body fluids to form a hydroxyapatite layer on the surface, was developed, and its ion release, pH variation, and apatite formation properties were examined. Over a period of 90 days, 50 wt.% BAG released 14,765 μg/L of calcium and 48 μg/L of phosphate, creating an alkaline environment with a pH of 8.9. Qualitative assessment also confirmed the formation of particles composed of calcium and phosphate on the surface of the 50 wt.% BAG specimens. The null hypothesis that the ion release, pH variation, and apatite-forming ability of the pit and fissure sealant containing 45S5 BAG would not be significantly different from those of the pit and fissure sealants without the 45S5 BAG filler was rejected. Consequently, the pit and fissure sealant containing 45S5 BAG can be used as a promising preventive dental material because it not only exhibits alkalinity, which can lower the risk of caries, but also releases Ca and P ions for a long time and forms apatite on the surface of the material.

## Figures and Tables

**Table 1:** Compositions of filler proportions in the experimental groups (wt.%).

Group	Resin Matrix (wt.%)	Filler (wt.%)	
		45S5 BAG	Silanized Glass Filler
0 wt.% BAG	50	0	50.0
12.5 wt.% BAG	50	12.5	37.5
25 wt.% BAG	50	25.0	25.0
37.5 wt.% BAG	50	37.5	12.5
50 wt.% BAG	50	50.0	0

## Data Availability

The data will be made available by the corresponding author upon reasonable request due to privacy restrictions.

## References

[1] Ortiz A.S., Tomazoni F., Knorst J.K., Ardenghi T.M. (2020). Influence of socioeconomic inequalities on levels of dental caries in adolescents: A cohort study. Int. J. Paediatr. Dent..

[2] Lam P.P.Y., Sardana D., Ekambaram M., Lee G.H.M., Yiu C.K.Y. (2020). Effectiveness of pit and fissure sealants for preventing and arresting occlusal caries in primary molars: A systematic review and meta-analysis. J. Evid. Based Dent. Pract..

[3] Rogers H.J., Gilchrist F., Marshman Z., Rodd H.D., Rowen D. (2020). Selection and validation of a classification system for a child-centred preference-based measure of oral health-related quality of life specific to dental caries. J. Patient Rep. Outcomes.

[4] Gilchrist F., Marshman Z., Deery C., Rodd H.D. (2015). The impact of dental caries on children and young people: What they have to say?. Int. J. Paediatr. Dent..

[5] Dimaisip-Nabuab J., Duijster D., Benzian H., Heinrich-Weltzien R., Homsavath A., Monse B., Sithan H., Stauf N., Susilawati S., Kromeyer-Hauschild K. (2018). Nutritional status, dental caries and tooth eruption in children: A longitudinal study in Cambodia, Indonesia and Lao PDR. BMC Pediatr..

[6] Chabadel O., Véronneau J., Montal S., Tramini P., Moulis E. (2021). Effectiveness of pit and fissure sealants on primary molars: A 2-yr split-mouth randomized clinical trial. Eur. J. Oral Sci..

[7] Choi J.W., Yang S.Y. (2023). Effect of zinc oxide incorporation on the antibacterial, physicochemical, and mechanical properties of pit and fissure sealants. Polymers.

[8] Memarpour M., Abedinzade A., Rafiee A., Hashemian A. (2022). Penetration ability and microhardness of infiltrant resin and two pit and fissure sealants in primary teeth with early enamel lesions. Sci. Rep..

[9] AlQahtani A., Al-Dlaigan Y., Almahdy A. (2022). Microtensile bond strength of bioactive pit and fissure sealants bonded to primary and permanent teeth. Materials.

[10] Wright J.T., Crall J.J., Fontana M., Gillette E.J., Nový B.B., Dhar V., Donly K., Hewlett E.R., Quinonez R.B., Chaffin J. (2016). Evidence-based clinical practice guideline for the use of pit-and-fissure sealants: A report of the American Dental Association and the American Academy of Pediatric Dentistry. J. Am. Dent. Assoc..

[11] Ozan G., Sancakli H.S., Erdemir U., Yaman B.C., Yildiz S.O., Yildiz E. (2022). Comparative evaluation of a fissure sealant and a flowable composite: A 36-month split-mouth, randomized clinical study. J. Dent..

[12] Kaga M., Kakuda S., Ida Y., Toshima H., Hashimoto M., Endo K., Sano H. (2014). Inhibition of enamel demineralization by buffering effect of S-PRG filler-containing dental sealant. Eur. J. Oral Sci..

[13] Yang S.Y., Choi J.W., Kim K.M., Kwon J.S. (2020). Prevention of secondary caries using resin-based pit and fissure sealants containing hydrated calcium silicate. Polymers.

[14] Lee M.J., Mangal U., Kim S.-J., Yoon Y.-P., Ahn E.-S., Jang E.-S., Kwon J.-S., Choi S.-H. (2020). Improvement in the microbial resistance of resin-based dental sealant by sulfobetaine methacrylate incorporation. Polymers.

[15] Huang Y., Li H., Zhu C.G., Zhou X., Wang H., Han Q., Ren B., Cheng L. (2021). Anti-bacterial and anti-microbial aging effects of resin-based sealant modified by quaternary ammonium monomers. J. Dent..

[16] Fei X., Li Y., Weir M.D., Baras B.H., Wang H., Wang S., Sun J., Melo M.A., Ruan J., Xu H.H. (2020). Novel pit and fissure sealant containing Nano-CaF2 and dimethylaminohexadecyl methacrylate with double benefits of fluoride release and antibacterial function. Dent. Mater..

[17] Swetha D.L., Vinay C., Uloopi K.S., Rojaramya K.S., Chandrasekhar R. (2019). Antibacterial and mechanical properties of pit and fissure sealants containing zinc oxide and calcium fluoride nanoparticles. Contemp. Clin. Dent..

[18] Memarpour M., Afzali Baghdadabadi N., Rafiee A., Vossoughi M. (2020). Ion release and recharge from a fissure sealant containing amorphous calcium phosphate. PLoS ONE.

[19] Unal M., Oztas N. (2015). Remineralization capacity of three fissure sealants with and without gaseous ozone on non-cavitated incipient pit and fissure caries. J. Clin. Pediatr. Dent..

[20] Raszewski Z., Chojnacka K., Mikulewicz M. (2022). Preparation and characterization of acrylic resins with bioactive glasses. Sci. Rep..

[21] Baino F., Hamzehlou S., Kargozar S. (2018). Bioactive glasses: Where are we and where are we going?. J. Funct. Biomater..

[22] Ma Q., Chen J., Xu X., Wang T. (2020). Impact of transparent tray-based application of bioactive glasses desensitizer on the permeability of enamel and dentin to hydrogen peroxide: An in vitro study. BMC Oral Health.

[23] Yang S.Y., Han A.R., Kim K.M., Kwon J.S. (2022). Effects of incorporating 45S5 bioactive glass into 30% hydrogen peroxide solution on whitening efficacy and enamel surface properties. Clin. Oral Investig..

[24] Yang S.Y., Kim S.H., Choi S.Y., Kim K.M. (2016). Acid neutralizing ability and shear bond strength using orthodontic adhesives containing three different types of bioactive glass. Materials.

[25] Yang S.Y., Kwon J.S., Kim K.N., Kim K.M. (2016). Enamel surface with pit and fissure sealant containing 45S5 bioactive glass. J. Dent. Res..

[26] (2021). Biological Evaluation of Medical Devices—Part 12: Sample Preparation and Reference Materials.

[27] Al-Eesa N.A., Johal A., Hill R.G., Wong F.S.L. (2018). Fluoride containing bioactive glass composite for orthodontic adhesives apatite formation properties. Dent. Mater..

[28] Flores-Ledesma A., Tejeda-Cruz A., Bucio L., Wintergerst A.M., Rodríguez-Chávez J.A., Moreno-Vargas Y.A., Arenas-Alatorre J.A. (2020). Hydration products and bioactivity of an experimental MTA-like cement modified with wollastonite and bioactive glass. Ceram. Int..

[29] Westhauser F., Hohenbild F., Arango-Ospina M., Schmitz S.I., Wilkesmann S., Hupa L., Moghaddam A., Boccaccini A.R. (2020). Bioactive glass (BG) ICIE16 shows promising osteogenic properties compared to crystallized 45S5-BG. Int. J. Mol. Sci..

[30] Baheiraei N., Eyni H., Bakhshi B., Najafloo R., Rabiee N. (2021). Effects of strontium ions with potential antibacterial activity on in vivo bone regeneration. Sci. Rep..

[31] Erasmus E.P., Johnson O.T., Sigalas I., Massera J. (2017). Effects of sintering temperature on crystallization and fabrication of porous bioactive glass scaffolds for bone regeneration. Sci. Rep..

[32] Par M., Mohn D., Attin T., Tarle Z., Tauböck T.T. (2020). Polymerization shrinkage behaviour of resin composites functionalized with unsilanized bioactive glass fillers. Sci. Rep..

[33] Bakry A.S., Abbassy M.A. (2019). The efficacy of a bioglass (45S5) paste temporary filling used to remineralize enamel surfaces prior to bonding procedures. J. Dent..

[34] Rojas O., Prudent M., López M.E., Vargas F., Ageorges H. (2020). Influence of atmospheric plasma spraying parameters on porosity formation in coatings manufactured from 45S5 bioglass^®^ powder. J. Therm. Spray Technol..

[35] Yang S.Y., Piao Y.-Z., Kim S.-M., Lee Y.-K., Kim K.-N., Kim K.-M. (2013). Acid neutralizing, mechanical and physical properties of pit and fissure sealants containing melt-derived 45S5 bioactive glass. Dent. Mater..

[36] Xie X., Wang L., Xing D., Qi M., Li X., Sun J., Melo M.A.S., Weir M.D., Oates T.W., Bai Y. (2019). Novel rechargeable calcium phosphate nanoparticle-filled dental cement. Dent. Mater. J..

[37] Lehmann A., Nijakowski K., Drożdżyńska A., Przybylak M., Woś P., Surdacka A. (2022). Influence of the polymerization modes on the methacrylic acid release from dental light-cured materials-in vitro study. Materials.

[38] Juntavee N., Juntavee A., Plongniras P. (2018). Remineralization potential of nano-hydroxyapatite on enamel and cementum surrounding margin of computer-aided design and computer-aided manufacturing ceramic restoration. Int. J. Nanomed..

[39] Jang J.H., Kim H.-J., Choi J.-Y., Kim H.-W., Choi S., Kim S., Bang A., Kim D.-S. (2022). Effect of dentin desensitizer containing novel bioactive glass on the permeability of dentin. Materials.

[40] Liaqat S., Aljabo A., Khan M.A., Ben Nuba H., Bozec L., Ashley P., Young A. (2015). Characterization of dentine to assess bond strength of dental composites. Materials.

[41] Kim H.J., Jang J.-H., Woo S.U., Choi K.-K., Kim S.-Y., Ferracane J.L., Lee J.-H., Choi D., Choi S., Kim S. (2021). Effect of novel bioactive glass-containing dentin adhesive on the permeability of demineralized dentin. Materials.

[42] Cancelliere R., Rea G., Micheli L., Mantegazza P., Bauer E.M., El Khouri A., Tempesta E., Altomare A., Capelli D., Capitelli F. (2023). Electrochemical and structural characterization of lanthanum-doped hydroxyapatite: A promising material for sensing applications. Materials.

[43] Yang S.Y., Han A.R., Choi J.W., Kim K.M., Kwon J.S. (2023). Novel antibacterial and apatite forming restorative composite resin incorporated with hydrated calcium silicate. Biomater. Res..

[44] Beun S., Bailly C., Devaux J., Leloup G. (2012). Physical, mechanical and rheological characterization of resin-based pit and fissure sealants compared to flowable resin composites. Dent. Mater..

